# GASTRIC RESERVOIR NECROSIS POST GASTRO-JEJUNAL BYPASS. THE IMPORTANCE OF CLINICAL EVALUATION IN THE DECISION MAKING PROGRESS: CASE REPORT

**DOI:** 10.1590/0102-6720201600S10034

**Published:** 2016

**Authors:** Manuel ACEVES Avalos, Erik Ivan BARRAGÁN Veloz, Humberto ARENAS Marquez, Raúl PÉREZ Gomez, Arturo MARTINEZ Medrano, Eduardo Daniel ACEVES Velazquez, Enrique VARGAS Maldonado, Edgar CASTILLO Salas

**Affiliations:** 1 Obesidad y Laparoscopia Avanzada (OLA), Hospital Puerta de Hierro Sur, Guadalajara, Jalisco, México; 2 Internal Medicine, Hospital San José Tec de Monterrey, Monterrey, Nuevo León, México

**Keywords:** Roux-en-Y gastric bypass, Necrosis gastric pouch, Esophagojejunal anastomosis

## INTRODUCTION

Obesity is the major epidemic of our generation^8^. In Mexico statistics are impressive, and alarming. According to The Organization for Economic Co-operation and Development (OECD) places the country in the first quintile of the obesity distribution in America^1^. A growing number of patients are undergoing surgical treatment for their morbid obesity. Laparoscopic Roux-en-Y gastric bypass (LRYGB) is a technically challenging procedure with a steep learning curve. It gives the best long-lasting excess weight loss, but is a challenging procedure with potential life-threatening complications^11^.

The surgical technique and experience in bariatric procedures continue advancing. Yet more complications continue to be a challenge for diagnosis and treatment. One of the most serious is early postoperative leak with resulting peritonitis. Many case reports and series have reported in the literature with successful early repair, uncomplicated postoperative leaks, and their management is well exposed^10^.

However, make a diagnostic and management of a necrotic gastric pouch has been rarely reported, the treatment options are not clearly delineated in the literature. In this paper is presented an early diagnostic and management strategy for reestablishing the continuity of gastrointestinal tract following an LRYGB procedure complicated with gastric pouch necrosis. The operative management had result in resect the reservoir and performing a primary esophagojejunal anastomosis. The key for the management of this complication is its detection based primarily in clinical suspicion and early treatment. 

## CASE REPORT

**FIGURE 1:**
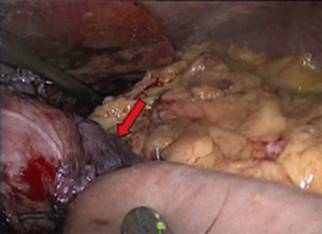
Gastrojejunal anastomosis - first surgery: RYGB, it seems to be a little ischemic area at the gastric pouch which was covered with omentum

**FIGURE 2:**
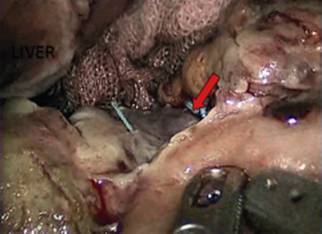
Gastrojejunal anastomosis - second surgery: the ischemic area is bigger than the last surgery

**FIGURE 3:**
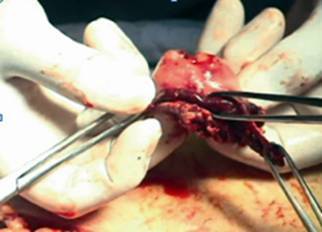
Third surgery: necrotic pouch

This is the case of a 38 year old woman with previous history of adjustable gastric band removal for band reservoir infection five years earlier that underwent LRYGBP and presented 48 h later with tachycardia, tachypnea and a drop in hemoglobin. The team decides to surgically explore her, finding hemoperitoneum with a minor leak on the anterior aspect of the gastric reservoir (Figure 1). The hematic content was drained, the bleeding site sutured and a fibrin seal was put in, leaving a drain in place. The patient improved clinically over the next 48 h to then present dark, foul liquid through the drain accompanied by halitosis like the drain liquid, tachycardia and tachypnea. The team decides to re-explore her, finding the same liquid without leak and a violet gastric reservoir (Figure 2). Was decided to resect the reservoir performing a primary esophagojejunal anastomosis, lavage and jejunostomy (Figures 3 and ^4^). A Wittmann patch was put in place for a schedule in advance re-exploration. The patient evolved satisfactorily. The new exploration revealed no leaks. A 5^th^ day exploration did not show anything relevant and was proceeded a definitive closure. The patient was discharged two weeks later. Pathology report was: Gastric reservoir necrosis secondary to massive venous thrombosis.

**FIGURE 4:**
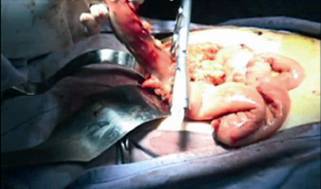
Section of the proximal jejunum

## DISCUSSION

The LRYGB is a combine bariatric procedure restrictive-malabsorptive, which allows a persistent weight loss^7^. It is a complex procedure and requires high surgical laparoscopic skill by the surgeon. One of the most important objectives in the early postoperative management of the gastric bypass patient is the prompt diagnosis and management of complications. Patients present early in the postoperative period with symptoms that vary from subtle (anxiety and mild tachycardia) to more evident (sepsis)^4^. 

While uncommon, frank necrosis of the gastric pouch following LRYGB is a life-threatening complication. Immediate operative management of this complication includes resection of the necrotic gastric pouch as well as diversion and drainage, and restore the continuity of the digestive tract. Brian K. Rundall et al.^3^ publish a case necrosis of the gastric pouch following LRYGB; their management consisted in performed a diverting cervical end esophagostomy to completely exclude the esophagus, secondary to abdominal sepsis. This effectively ruled out use of the esophagus in reconstruction of gastrointestinal continuity at a later operation. Using the stomach as conduit of choice to replace the esophagus was done taking good result^3^ ^,^ ^9^.

Marina Andres et al.^6^ described several factors that may be the trigger necrosis: obstruction at enteroenterostomy due to edema, adhesions, or even internal hernias causing distention and gastric, pancreatobiliary limb or jejunal wall necrosis^2^ ^,^ ^6^.

Jean-Marc Chevallier et al.^5^ describe their complications experience at 1000 patients at after laparoscopic adjustable gastric banding for morbid obesity, in which they present one case of gastric necrosis as a late complication; it was resolved by a total gastrectomy^5^. 

The gastric tissue is posteriorly fibrotic to a placement and removal of a gastric band; subsequent surgeries performed on the same gastric tissue slightly increases the risk of complications such as bleeding, leakage and ischemia. As in the this case report prior history of gastric banding was of clinical importance. 

Gastric pouch necrosis is a rare complication of LRYGB, and the resulting esophageal discontinuity can be challenging to correct. We decided to resect the reservoir performing a primary esophagojejunal anastomosis, lavage and a jejunostomy. The patient's progress was satisfactory, and was discharged two weeks later. The pathological study of the gastric pouch excised tissue showed the cause secondary to massive venous thrombosis. There is little information in the literature of management of necrosis of gastric pouch after LRYGB; the use the jejunum to restore the continuity of the digestive tract seems to be a safe and good option. 

The clinical evaluation of the patient must be the keystone that takes the team to an early exploration. This case is a clear and demonstrative example, allowing the resection and primary anastomosis without any major complication. This should always be the approach in these patients. The surgeon should not rely on laboratory or radiodiagnostics to decide whether or not to re-explore a patient.

## References

[1] Palloni Alberto, Beltrán-Sánchez Hiram, Novak Beatriz, Pinto Guido, Wong Rebeca (2015). Adult obesity, disease and longevity in México. Salud Publica Mex.

[2] Albuquerque Andreia (2012). Gastric necrosis caused by gastric banding.

[3] Brian K., Rundall D.O., Chadrick E Denlinger (2005). Laparoscopic Gastric Bypass Complicated by Gastric Pouch Necrosis: Considerations in Gastroesophageal Reconstruction. J Gastrointestinal Surgery.

[4] Byrne TK (2001). Complications of surgery for obesity. Surg Clin North Am.

[5] Chevallier Jean-Marc, Zinzindohoué Franck (2004). Complications after Laparoscopic Adjustable Gastric Banding for Morbid Obesity: Experience with 1,000 Patients over 7 Years Obesity. Surgery.

[6] Andres Marina, Perez Marta, Roldan Jose (2007). Roux-en-Y gastric bypass: major complications. Abdom Imaging.

[7] Merkle EM (2005). Roux-en-Y gastric bypass for clinically severe obesity: normal appearance and spectrum of complications at imaging. Radiology.

[8] Ogden CL, Carroll MD, Kit BK, Flegal KM (2012). Prevalence of obesity and trends in body mass index among US children and adolescents, 1999-2010. JAMA.

[9] Griffith P. Sahle (2012). Managing complications associated with laparoscopic Roux-in-Y gastric bypass for morbid Obesity. Can J Surg.

[10] Papasavas PK, Caushaj PF, McCormick JT (2003). Laparoscopic management of complications following laparoscopic Roux-en-Y gastric bypass for morbid obesity. Surg Endosc.

[11] Westling A, Gustavsson S (2001). Laparoscopic vs open Roux-en-Y gastric bypass: a prospective, randomized trial. Obes Surg.

